# Global temporal trends and projections of hepatitis B-related cirrhosis among adolescents and young adults from 1990 to 2035: an analysis based on the global burden of disease study 2021

**DOI:** 10.3389/fpubh.2024.1494388

**Published:** 2025-01-08

**Authors:** Quanwei He, Xiujuan Chang, Ran Xu, Wei Han, Sihao Wang, Shujuan Gong, Jiagan Huang, Jiangtao Liu, Rugang Zhang, Yongping Yang

**Affiliations:** ^1^Department of Gastroenterology, Hainan Hospital of Chinese PLA General Hospital, Sanya, China; ^2^Medical School of Chinese PLA, Beijing, China; ^3^Department of Hepatology, The Fifth Medical Center of Chinese PLA General Hospital, Beijing, China

**Keywords:** hepatitis B, cirrhosis, adolescents, young adults, incidence, mortality, disability adjusted-life years, GBD 2021

## Abstract

**Background:**

Chronic hepatitis B and cirrhosis pose significant global health threats. Few studies have explored the disease burden and mortality trend of cirrhosis caused by hepatitis B virus infection among adolescents and young adults (AYAs, aged 15–39 years). This study aimed to assess the disease burden and trends.

**Methods:**

Publicly available data were obtained from the 2021 GBD database. The rates of incidence, mortality, and disability-adjusted life years were calculated at the global, regional, and national levels. Temporal trends were assessed using joinpoint regression analysis, while the Bayesian age-period-cohort model was used to predict future trends.

**Results:**

From 1990 to 2021, the global incidence rate of hepatitis B-related cirrhosis decreased from 111.33 (95% uncertainty interval: 89.18 to 134.98) to 67.75 (54.06 to 82.71) per 100,000 with an average annual percentage change of −1.58 (95% confidence interval: −1.66 to −1.51, *p* < 0.001). However, between 1990 and 2021, the incidence numbers in the 30–34 and 35–39 age groups increased by 23.75 and 21.24%, respectively. The number of deaths in low and low-middle Socio-demographic Index (SDI) areas increased by 79.51 and 20.62%, respectively. Moreover, it is predicted that the numbers of incidences and deaths will continue to rise in areas with low SDI. At the regional level, Central Sub-Saharan Africa had the highest incidence and mortality rates. In 2021, Somalia and the Democratic Republic of Congo had the highest incidence rates, whereas Kiribati and Cambodia had the highest mortality rates.

**Conclusion:**

The overall burden of hepatitis B-related cirrhosis among AYAs has decreased over the past three decades. Nevertheless, there was a slight increase in the incidence number among individuals aged 30–39 years. The substantial burden and predicted rise in the numbers of incidences and deaths in low SDI areas underscore the need for sustained and targeted public health interventions.

## 1 Introduction

Hepatitis B virus (HBV) infection is a significant global public health challenge, imposing a substantial healthcare burden, particularly in the Western Pacific and African regions (1). Chronic hepatitis B (CHB) can lead to liver cirrhosis, which may progress to liver failure and hepatocellular carcinoma (HCC), posing a serious threat to human health (2, 3). A comprehensive analysis of the global, regional, and national burden of hepatitis B between 1990 and 2019 revealed a decline in HBV prevalence across all age groups, with a significant reduction in children (1). Despite notable advancements in prevention and treatment, hepatitis B remains the leading cause of liver-related incidence and mortality. According to the World Health Organization (WHO), hepatitis B accounted for estimated 1.1 million deaths in 2022, primarily due to cirrhosis and HCC (4). Additionally, the number of deaths related to HBV has gradually increased globally, with cirrhosis being the predominant cause of mortality (5).

CHB is often acquired during childhood and may progress over decades (6). Previous studies identified age as a critical risk factor for developing cirrhosis and HCC. For instance, a study on the natural history of CHB indicated that individuals over 40 years old are at a higher risk of developing cirrhosis (7). Advancing age was independently associated with the development of cirrhosis and HCC in patients with CHB, particularly in Asian populations (6, 8). However, recent studies have highlighted a potential increase in cirrhosis burden among younger individuals (9). Additionally, a cohort study demonstrated that liver fibrosis scores were significantly associated with an increased risk of liver-related mortality regardless of age group (<40 vs. ≥40 years) (10). This underscores the importance of considering younger patients under 39 years old a population at risk for cirrhosis and HCC.

Adolescents and young adults (AYAs), ranging from approximately 15–39 years of age, are the driving force of social development (11). Recently, some studies, respectively, reported the disease burden and trends changes of acute viral hepatitis and other chronic liver diseases such as alcoholic liver disease, and NAFLD in AYAs worldwide from 1990 to 2019 (12–14). Despite the extensive research conducted on the global burden of hepatitis B and cirrhosis (1, 9), few studies have explored the disease burden and mortality trend of cirrhosis caused by HBV infection among AYAs.

The Global Burden of Disease (GBD) study offers a comprehensive framework for understanding the epidemiology of hepatitis B-related cirrhosis. The GBD 2021 database contains extensive data on disease incidence, mortality, and risk factors across 204 countries and regions from 1990 to 2021 (15). In this study, we utilized GBD 2021 data to systematically analyze the epidemiological characteristics and temporal trends of hepatitis B-related cirrhosis among AYAs using joinpoint regression analysis (16) for the first time. Furthermore, we applied the Bayesian age-period-cohort (BAPC) analysis (17) to predict the rate and number of hepatitis B-related cirrhosis cases from 2022 to 2035.

## 2 Materials and methods

### 2.1 Data sources

The data for this study were sourced from the GBD 2021 database, which is publicly available and provided by the Institute for Health Metrics and Evaluation at the University of Washington, USA.^1^ This database contains information on the incidence, mortality, and 88 risk factors for 371 diseases and injuries across 204 countries and territories globally from 1990 to 2021 (15). Data on the disease burden data of CHB including cirrhosis among AYAs (15–39 years) were extracted using the GBD Results tool.^2^ Cirrhosis was diagnosed based on the International Classification of Diseases, 10th edition, as described in earlier GBD studies (18, 19). All rates were standardized per 100,000 population and accompanied by 95% uncertainty intervals (UIs).

### 2.2 Sociodemographic index (SDI)

The SDI is a composite measure of a country’s sociodemographic development, incorporating income per capita, educational attainment, and total fertility rate. Based on the 2021 SDI values (Supplementary Table S1), countries and regions were classified into five levels: low, low-middle, middle, middle-high, and high SDI (20).

### 2.3 Joinpoint regression analysis

The joinpoint regression model has been widely applied in analyzing trends in disease burden changes (16). This model included both linear models and log-linear models. In this study, we selected log-linear models to assess the trends of incidence, mortality, and disability-adjusted life years (DALYs) over time (21, 22). The number and location of joinpoints were determined using the grid search method, with a maximum limit of 5 joinpoints. Model optimization was performed using Monte Carlo permutation tests. The annual percentage change rate (APC) and the average annual percentage change rate (AAPC) were calculated along with 95% confidence intervals (CIs). The specific calculation methods have been reported in previous studies (19).

### 2.4 BAPC analysis

The BAPC and integrated nested Laplacian approximation (INLA) packages (version 4.1.0) were used to predict trends in hepatitis B-related cirrhosis among AYAs from 2022 to 2035. The BAPC model, which builds upon INLA, provides reliable estimates of the marginal posterior distributions, demonstrating superior predictive performance compared to other models (17, 23, 24). The specific calculation methods have been reported in previous studies (25). Based on historical and demographic data, we have projected global incidence and mortality rates and numbers at different SDI levels, providing 95% CIs for these predictions.

### 2.5 Statistical analysis

All analyses and visualizations were conducted using R software (version 4.3.2). Joinpoint software (version 4.8.0)^3^ was used for trend analysis. Two-tailed *p*-values of less than 0.05 were considered statistically significant.

## 3 Results

### 3.1 The global incidence, mortality, and DALYs rates of hepatitis B-related cirrhosis among AYAs from 1990 to 2021

**Figure 1:**
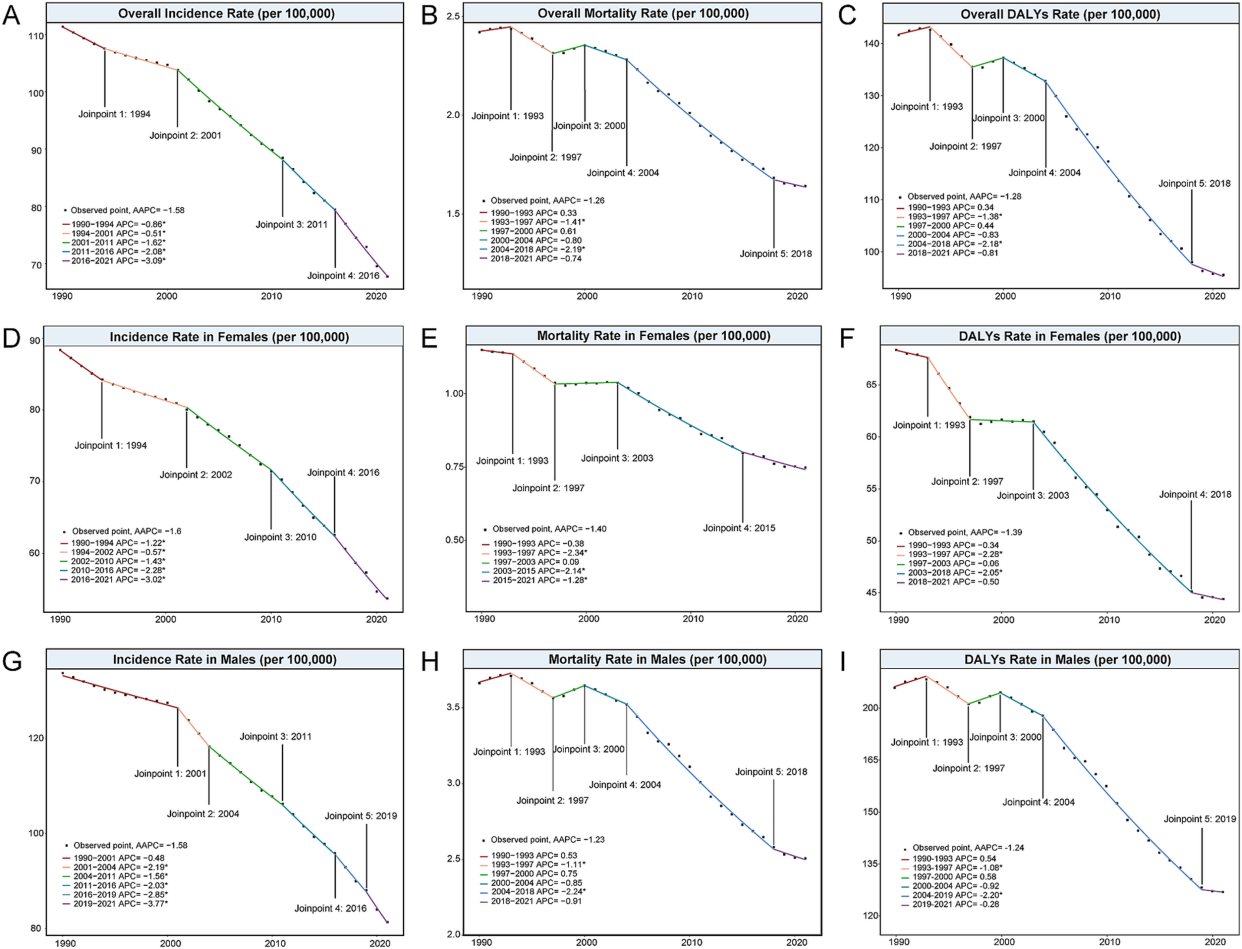
Joinpoint analysis of the global incidence, mortality, and DALYs rates (per 100,000 population) of Hepatitis B-related cirrhosis among AYAs from 1990 to 2021. **(A)** Overall incidence rate; **(B)** Overall mortality rate; **(C)** Overall DALYs rate; **(D)** Incidence rate in females; **(E)** Mortality rate in females; **(F)** DALYs rate in females; **(G)** Incidence rate in males; **(H)** Mortality rate in males; **(I)** DALYs rate in males. AYAs, Adolescents and Young Adults; DALYs, disability-adjusted life years.

From 1990 to 2021, the global incidence rate of hepatitis B-related cirrhosis decreased from 111.33 per 100,000 (95%UI: 89.18 to 134.98) to 67.75 per 100,000 (95% UI: 54.06 to 82.71) (Table 1). The global AAPC of incidence was −1.58 [95% confidence interval (CI): −1.66 to −1.51, *p* < 0.001] (Table 2), indicating a significant decline over the 31-year period. In addition, the joinpoint regression model identified significant changes in incidence rates in 1994, 2001, 2011, and 2016, respectively (Figure 1A and Supplementary Table 2). Despite the overall decrease in the incidence rate, the incidence number increased in the 30–34 and 35–39 age groups, reflecting a shift in disease burden to older young adults (Table 1). The global number and rate of deaths from hepatitis B-related cirrhosis among AYAs showed a slight decline between 1990 and 2021 (Table 1). The AAPC of DALYs also decreased (AAPC = −1.28 [95% CI: −1.53 to −1.03], *p* < 0.001) (Table 2), illustrating a reduction in the overall disease burden. Notably, the joinpoint model showed a “rising-falling-rising-falling again” trend in the global DALYs rate, with a similar trend in the mortality rate from 1990 to 2021 (Figures 1B,C and Supplementary Tables 3, 4).

**Table 1:** Incidence and mortality of Hepatitis B-related cirrhosis among AYAs by gender, age, SDI, and region in 1990 and 2021.

Subgroup	1990		2021			1990		2021		
	Incidence number × 10^3^ (95% UI)	Incidence rate per 100,000 (95% UI)	Incidence number × 10^3^ (95% UI)	Incidence rate per 100,000 (95% UI)	Change of number (%)	Number of deaths × 10^3^ (95% UI)	Mortality rate per 100,000 (95% UI)	Number of deaths × 10^3^ (95% UI)	Mortality rate per 100,000 (95% UI)	Change of number (%)
Global	2440.06 (1954.60–2958.52)	111.33 (89.18–134.98)	2015.39 (1608.22–2460.41)	67.75 (54.06–82.71)	−17.40	53.06 (44.24–63.74)	2.42 (2.02–2.91)	48.82 (39.05–59.31)	1.64 (1.31–1.99)	−7.99
Gender										
Male	1482.62 (1227.27–1780.10)	133.75 (110.72–160.59)	1228.63 (1006.72–1488.25)	81.38 (66.68–98.58)	−17.13	40.61 (33.47–49.01)	3.66 (3.02–4.42)	37.86 (30.38–46.18)	2.51 (2.01–3.06)	−6.78
Female	957.44 (729.71–1201.10)	88.38 (67.36–110.87)	786.76 (599.50–980.14)	53.70 (40.92–66.90)	−17.83	12.45 (9.76–15.56)	1.15 (0.90–1.44)	10.97 (8.42–13.86)	0.75 (0.57–0.95)	−11.94
Age groups										
15–19 years	671.20 (533.44–828.94)	129.22 (102.70–159.59)	317.77 (252.92–391.44)	50.93 (40.53–62.73)	−52.66	1.68 (1.11–2.54)	0.32 (0.21–0.49)	1.04 (0.58–1.74)	0.17 (0.09–0.28)	−38.33
20–24 years	580.31 (447.43–722.12)	117.93 (90.93–146.75)	378.73 (296.48–470.86)	63.42 (49.65–78.85)	−34.74	4.68 (3.45–6.07)	0.95 (0.70–1.23)	3.70 (2.44–5.30)	0.62 (0.41–0.89)	−21.09
25–29 years	475.37 (374.20–582.50)	107.40 (84.54–131.60)	444.78 (347.39–548.98)	75.60 (59.05–93.31)	−6.43	8.78 (6.74–11.24)	1.98 (1.52–2.54)	7.88 (5.60–10.59)	1.34 (0.95–1.80)	−10.25
30–34 years	378.46 (299.64–465.07)	98.19 (77.74–120.67)	468.34 (364.81–581.09)	77.48 (60.35–96.13)	23.75	14.55 (11.95–17.75)	3.78 (3.10–4.61)	14.49 (11.44–18.12)	2.40 (1.89–3.00)	−0.44
35–39 years	334.70 (254.73–419.39)	95.02 (72.32–119.06)	405.78 (308.75–518.71)	72.35 (55.05–92.48)	21.24	23.37 (18.83–29.04)	6.64 (5.34–8.24)	21.73 (16.45–27.61)	3.87 (2.93–4.92)	−7.04
SDI-levels										
Low SDI	279.91 (213.58–346.24)	151.87 (115.88–187.86)	552.82 (436.85–675.03)	123.11 (97.28–150.32)	97.50	6.00 (4.75–7.68)	3.25 (2.58–4.17)	10.76 (8.23–13.84)	2.40 (1.83–3.08)	79.51
Low-middle SDI	423.94 (340.20–518.11)	93.50 (75.03–114.27)	529.57 (432.93–637.80)	65.99 (53.95–79.48)	24.91	14.31 (10.98–18.34)	3.16 (2.42–4.04)	17.27 (13.03–21.58)	2.15 (1.62–2.69)	20.62
Middle SDI	1025.24 (816.91–1247.09)	136.22 (108.54–165.70)	622.09 (492.82–765.24)	67.07 (53.13–82.51)	−39.32	21.71 (18.64–25.17)	2.88 (2.48–3.34)	15.12 (12.49–18.17)	1.63 (1.35–1.96)	−30.35
High-middle SDI	537.29 (429.85–656.47)	118.73 (94.99–145.06)	237.35 (175.71–304.85)	53.91 (39.91–69.24)	−55.82	8.26 (6.98–9.60)	1.83 (1.54–2.12)	4.50 (3.60–5.49)	1.02 (0.82–1.25)	−45.55
High SDI	172.24 (147.13–202.90)	49.64 (42.40–58.48)	72.44 (59.32–88.57)	20.51 (16.79–25.07)	−57.94	2.75 (2.18–3.39)	0.79 (0.63–0.98)	1.15 (0.88–1.47)	0.32 (0.25–0.42)	−58.29
Regions										
Andean Latin America	6.09 (5.03–7.62)	39.39 (32.55–49.27)	7.96 (6.74–9.77)	29.38 (24.91–36.07)	30.62	0.12 (0.07–0.18)	0.76 (0.47–1.18)	0.09 (0.05–0.15)	0.33 (0.20–0.56)	−23.38
Australasia	4.51 (3.30–5.85)	55.30 (40.53–71.78)	3.19 (2.39–4.09)	30.51 (22.87–39.05)	−29.16	0.03 (0.02–0.04)	0.34 (0.23–0.47)	0.02 (0.01–0.02)	0.17 (0.11–0.24)	−34.97
Caribbean	4.84 (3.61–6.28)	32.57 (24.30–42.23)	4.32 (3.25–5.45)	23.71 (17.84–29.91)	−10.85	0.09 (0.05–0.14)	0.61 (0.36–0.93)	0.09 (0.05–0.14)	0.48 (0.27–0.79)	−3.07
Central Asia	24.67 (20.07–28.64)	86.69 (70.53–100.65)	24.13 (19.67–28.09)	64.55 (52.62–75.13)	−2.16	0.27 (0.17–0.41)	0.95 (0.59–1.44)	0.57 (0.35–0.88)	1.52 (0.92–2.35)	110.42
Central Europe	23.92 (19.86–28.30)	51.05 (42.39–60.41)	7.39 (6.07–9.03)	21.10 (17.34–25.79)	−69.10	0.69 (0.46–0.96)	1.46 (0.98–2.04)	0.35 (0.25–0.48)	1.00 (0.71–1.37)	−48.91
Central Latin America	24.51 (16.91–34.21)	35.90 (24.77–50.11)	15.49 (9.91–21.65)	15.31 (9.80–21.40)	−36.78	19.20 (16.33–22.29)	0.46 (0.35–0.60)	7.68 (5.95–9.50)	0.27 (0.20–0.38)	−12.63
Central Sub-Saharan Africa	50.81 (35.66–67.52)	244.72 (171.75–325.20)	120.08 (88.56–152.48)	221.97 (163.70–281.87)	136.33	0.31 (0.24–0.41)	3.62 (2.32–5.09)	0.27 (0.20–0.39)	2.93 (1.84–4.32)	110.67
East Asia	1126.73 (895.23–1376.78)	199.17 (158.25–243.37)	443.59 (321.69–570.27)	92.60 (67.15–119.04)	−60.63	0.75 (0.48–1.06)	3.39 (2.89–3.94)	1.58 (1.00–2.34)	1.60 (1.24–1.98)	−60.00
Eastern Europe	23.74 (17.11–31.12)	27.68 (19.95–36.29)	11.96 (8.28–15.99)	18.07 (12.52–24.16)	−49.64	0.18 (0.13–0.25)	0.21 (0.16–0.29)	0.73 (0.52–1.02)	1.10 (0.79–1.54)	300.76
Eastern Sub-Saharan Africa	102.11 (75.84–128.36)	144.04 (106.99–181.06)	179.91 (137.40–225.41)	102.69 (78.43–128.67)	76.20	2.21 (1.70–2.83)	3.12 (2.39–3.99)	4.47 (3.34–5.86)	2.55 (1.91–3.34)	101.88
High-income Asia Pacific	63.23 (55.14–72.61)	93.68 (81.70–107.58)	22.83 (19.58–26.94)	45.16 (38.74–53.30)	−63.90	1.08 (0.74–1.34)	1.61 (1.09–1.99)	0.15 (0.11–0.20)	0.29 (0.21–0.40)	−86.45
High-income North America	16.51 (12.57–20.73)	14.57 (11.09–18.29)	8.47 (6.45–10.48)	6.87 (5.24–8.51)	−48.72	0.22 (0.17–0.29)	0.19 (0.15–0.26)	0.18 (0.13–0.24)	0.14 (0.11–0.20)	−18.87
North Africa and Middle East	117.09 (99.78–138.35)	87.49 (74.55–103.38)	115.84 (100.54–131.63)	45.56 (39.54–51.77)	−1.07	1.70 (1.23–2.26)	1.27 (0.92–1.69)	1.42 (0.92–2.04)	0.56 (0.36–0.80)	−16.41
Oceania	5.09 (3.62–6.55)	191.52 (136.16–246.71)	6.25 (4.60–8.09)	110.84 (81.58–143.55)	22.76	0.07 (0.04–0.10)	2.55 (1.66–3.75)	0.10 (0.07–0.14)	1.83 (1.31–2.40)	52.63
South Asia	299.03 (239.07–369.08)	69.28 (55.39–85.51)	424.05 (346.38–518.72)	53.61 (43.79–65.58)	41.81	13.93 (10.76–17.89)	3.23 (2.49–4.14)	17.97 (13.46–23.00)	2.27 (1.70–2.91)	29.01
Southeast Asia	262.80 (211.80–320.07)	133.40 (107.51–162.47)	222.18 (178.39–273.20)	80.11 (64.32–98.51)	−15.46	7.38 (5.66–9.87)	3.75 (2.87–5.01)	6.90 (5.18–9.01)	2.49 (1.87–3.25)	−6.55
Southern Latin America	3.31 (2.77–3.98)	17.36 (14.50–20.84)	3.44 (2.77–4.14)	13.35 (10.76–16.06)	3.96	0.12 (0.07–0.19)	0.63 (0.38–0.97)	0.06 (0.04–0.09)	0.23 (0.14–0.36)	−49.98
Southern Sub-Saharan Africa	28.16 (21.53–34.45)	130.28 (99.61–159.36)	23.42 (17.65–29.24)	68.80 (51.86–85.90)	−16.84	0.42 (0.33–0.53)	1.93 (1.50–2.45)	0.46 (0.31–0.63)	1.36 (0.90–1.85)	11.13
Tropical Latin America	37.66 (29.52–46.52)	58.56 (45.89–72.34)	18.27 (12.86–24.31)	20.69 (14.57–27.53)	−51.49	1.21 (1.05–1.38)	1.88 (1.63–2.14)	0.65 (0.55–0.77)	0.73 (0.62–0.87)	−46.40
Western Europe	44.10 (34.41–54.03)	30.60 (23.87–37.49)	18.24 (14.21–22.41)	14.05 (10.95–17.27)	−58.64	0.49 (0.32–0.72)	0.34 (0.22–0.50)	0.13 (0.08–0.20)	0.10 (0.06–0.15)	−73.65
Western Sub-Saharan Africa	171.15 (133.62–209.30)	239.13 (186.68–292.42)	334.41 (264.80–409.09)	174.89 (138.49–213.95)	95.39	2.61 (1.91–3.67)	3.64 (2.66–5.12)	4.96 (3.36–6.76)	2.59 (1.76–3.53)	90.32

AYAs, adolescents and young adults; SDI, Sociodemographic Index; UI, Uncertainty Interval.

**Table 2:** The AAPC of incidence, mortality, and DALYs from 1990 to 2021 by gender, SDI, region.

Subgroup	Incidence		Mortality		DALYs	
	AAPC (95% CI)	*p-*value	AAPC (95% CI)	*p-*value	AAPC (95% CI)	*p-*value
Global	−1.58 (−1.66, −1.51)	<0.001	−1.26 (−1.52, −1.00)	<0.001	−1.28 (−1.53, −1.03)	<0.001
Gender						
Male	−1.60 (−1.69, −1.51)	<0.001	−1.40 (−1.61, −1.20)	<0.001	−1.39 (−1.59, −1.19)	<0.001
Female	−1.58 (−1.70, −1.46)	<0.001	−1.23 (−1.54, −0.93)	<0.001	−1.24 (−1.55, −0.93)	<0.001
SDI-levels						
Low SDI	−0.66 (−0.71, −0.6)	<0.001	−1.00 (−1.08, −0.91)	<0.001	−0.99 (−1.07, −0.91)	<0.001
Low-middle SDI	−1.11 (−1.15, −1.07)	<0.001	−1.23 (−1.44, −1.03)	<0.001	−1.25 (−1.45, −1.04)	<0.001
Middle SDI	−2.27 (−2.41, −2.13)	<0.001	−1.85 (−2.08, −1.62)	<0.001	−1.90 (−2.17, −1.63)	<0.001
High-middle SDI	−2.54 (−2.69, −2.39)	<0.001	−1.91 (−2.34, −1.48)	<0.001	−1.96 (−2.40, −1.52)	<0.001
High SDI	−2.79 (−2.98, −2.60)	<0.001	−2.80 (−3.10, −2.50)	<0.001	−2.81 (−3.10, −2.53)	<0.001
Regions						
Andean Latin America	−0.95 (−1.01, −0.89)	<0.001	−2.63 (−3.32, −1.93)	<0.001	−2.65 (−3.34, −1.95)	<0.001
Australasia	−1.93 (−2.17, −1.69)	<0.001	−2.14 (−3.04, −1.22)	<0.001	−2.13 (−3.03, −1.22)	<0.001
Caribbean	−1.04 (−1.14, −0.95)	<0.001	−0.75 (−1.14, −0.37)	<0.001	−0.78 (−1.17, −0.38)	<0.001
Central Asia	−0.94 (−1.07, −0.82)	<0.001	1.40 (0.75, 2.05)	<0.001	1.31 (0.68, 1.94)	<0.001
Central Europe	−2.82 (−3.00, −2.64)	<0.001	−1.22 (−1.82, −0.62)	<0.001	−1.23 (−1.81, −0.64)	<0.001
Central Latin America	−2.73 (−2.91, −2.56)	<0.001	−1.66 (−1.97, −1.36)	<0.001	−1.67 (−1.97, −1.37)	<0.001
Central Sub-Saharan Africa	−0.28 (−0.35, −0.20)	<0.001	−0.73 (−0.89, −0.56)	<0.001	−0.73 (−0.90, −0.56)	<0.001
East Asia	−2.47 (−2.65, −2.30)	<0.001	−2.41 (−2.82, −2.00)	<0.001	−2.46 (−2.88, −2.04)	<0.001
Eastern Europe	−1.31 (−1.55, −1.06)	<0.001	5.50 (3.93, 7.09)	<0.001	5.39 (3.83, 6.96)	<0.001
Eastern Sub-Saharan Africa	−1.09 (−1.17, −1.01)	<0.001	−0.64 (−0.70, −0.58)	<0.001	−0.65 (−0.71, −0.60)	<0.001
High-income Asia Pacific	−2.33 (−2.58, −2.08)	<0.001	−5.36 (−5.67, −5.04)	<0.001	−5.35 (−5.64, −5.06)	<0.001
High-income North America	−2.38 (−2.57, −2.19)	<0.001	−0.82 (−1.32, −0.32)	<0.001	−0.82 (−1.29, −0.35)	<0.001
North Africa and Middle East	−2.10 (−2.18, −2.01)	<0.001	−2.64 (−2.91, −2.38)	<0.001	−2.62 (−2.81, −2.43)	<0.001
Oceania	−1.75 (−1.87, −1.64)	<0.001	−1.08 (−1.27, −0.88)	<0.001	−1.11 (−1.30, −0.91)	<0.001
South Asia	−0.81 (−0.86, −0.76)	<0.001	−1.15 (−1.54, −0.76)	<0.001	−1.16 (−1.54, −0.78)	<0.001
Southeast Asia	−1.60 (−1.72, −1.48)	<0.001	−1.36 (−1.48, −1.25)	<0.001	−1.41 (−1.53, −1.29)	<0.001
Southern Latin America	−0.94 (−1.12, −0.76)	<0.001	−3.15 (−4.42, −1.87)	<0.001	−3.12 (−4.36, −1.87)	<0.001
Southern Sub-Saharan Africa	−2.09 (−2.59, −1.59)	<0.001	−1.11 (−2.10, −0.12)	0.028	−1.14 (−2.10, −0.17)	0.021
Tropical Latin America	−3.33 (−3.51, −3.14)	<0.001	−3.02 (−3.23, −2.80)	<0.001	−3.06 (−3.27, −2.85)	<0.001
Western Europe	−2.48 (−2.62, −2.34)	<0.001	−3.98 (−4.36, −3.60)	<0.001	−3.97 (−4.35, −3.58)	<0.001
Western Sub-Saharan Africa	−1.00 (−1.04, −0.96)	<0.001	−1.11 (−1.25, −0.97)	<0.001	−1.11 (−1.24, −0.97)	<0.001

AAPC, average annual percentage changes; DALYs, disability-adjusted life-years; SDI, Sociodemographic Index; CI, confidence interval.

### 3.2 The incidence, mortality, and DALYs rates of hepatitis B-related cirrhosis by gender

Compared with 1990, both males and females experienced a reduction in the incidence number and rate of hepatitis B-related cirrhosis in 2021. Specifically, the incidence rate in males decreased from 133.75 per 100,000 to 81.38 per 100,000, while it declined from 88.38 per 100,000 to 53.70 per 100, 000 in females (Table 1). The incidence and mortality rates were higher in males than in females in both 1990 and 2021 (Supplementary Figures 1, 2). In addition, the AAPCs of incidence were −1.60 (95%CI: −1.69 to −1.51, *p* < 0.001) in males and −1.58 (95%CI: −1.70 to −1.46, *p* < 0.001) in females (Table 2), indicating a significant downward trend for both genders (Figures 1D,G). Between 1990 and 2021, the number of deaths decreased more in females (11.94%) than in males (6.78%) between 1990 and 2021 (Table 1). Notably, the joinpoint model suggested that the mortality and DALYs rates in males showed a “valley” in 1997, which differed from the trends in females (Figures 1E,F,H,I).

### 3.3 The incidence, mortality, and DALYs rates of hepatitis B-related cirrhosis by age

The incidence rate of hepatitis B-related cirrhosis across all age groups (15–39) decreased between 1990 and 2021 (Table 1 and Supplementary Figure 3). In particular, the most significant decline was observed in the 15–19 years, with the incidence rate dropping to 50.93 per 100,000 (95%UI: 40.53 to 62.73) and a 52.66% reduction in incidence number by 2021 (Table 1). In contrast, the incidence number increased by 23.75% in the 30–34 age group and by 21.24% in the 35–39 age group between 1990 and 2021 (Table 1). Furthermore, the incidence rate in the 30–34 age group was the highest among AYAs in 2021, at 77.48 per 100,000 (95%UI: 60.35 to 96.13) (Table 1). Despite an overall reduction in both the number and rate of deaths across all age groups, there remains concern regarding the mortality rate (3.87 per 100,000 [95%UI: 2.93 to 4.92]) and the number of deaths (21.73 × 10^3^ [95%UI: 16.45 × 10^3^ to 27.61 × 10^3^]) in the 35–39 age group, which remains to be the highest among AYAs (Table 1).

### 3.4 The incidence, mortality, and DALYs rates of hepatitis B-related cirrhosis in 204 countries and territories

**Figure 2:**
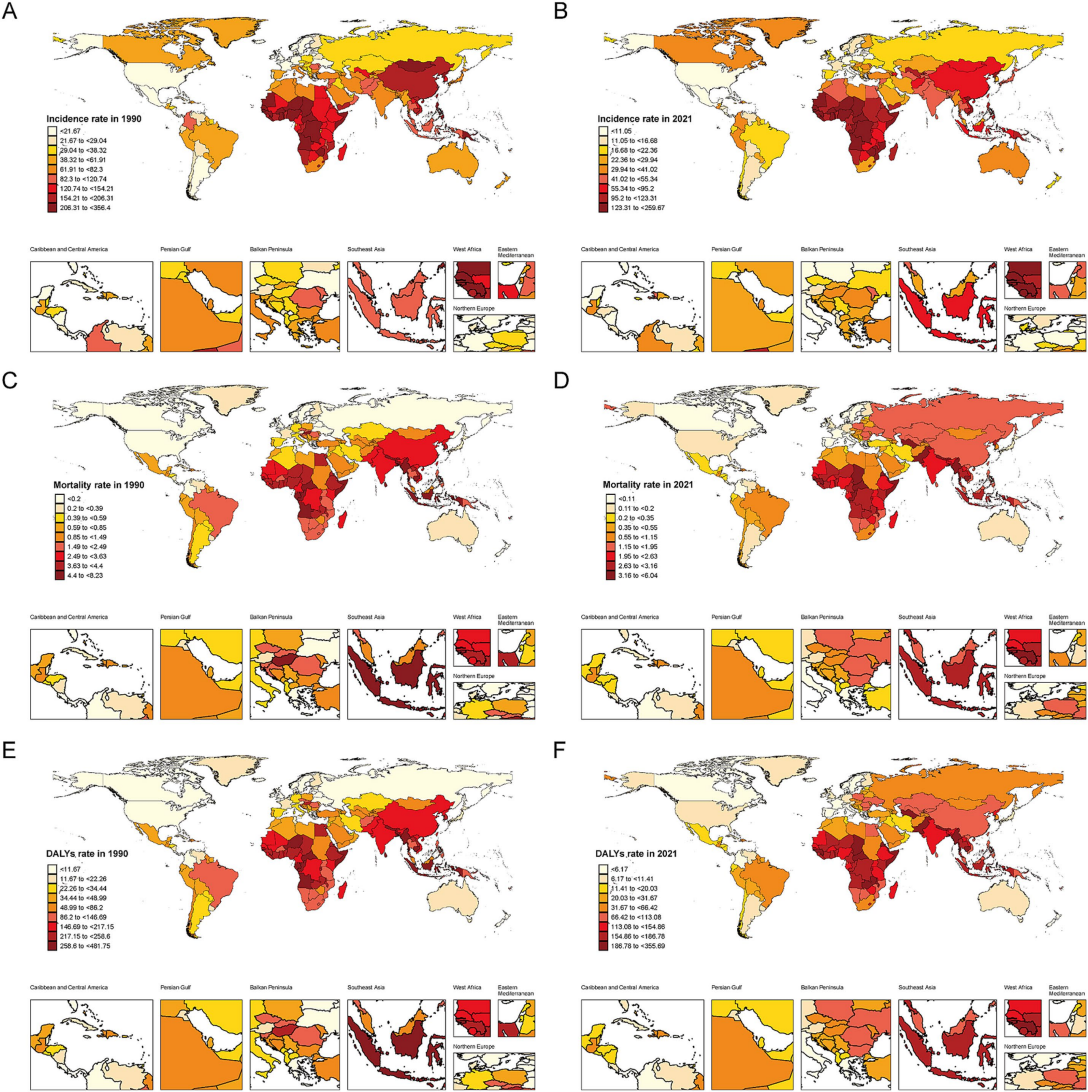
Geographical distribution of Hepatitis B-related cirrhosis among AYAs in 204 countries and territories. Darker colors indicate higher rates. **(A)** Incidence rate (per 100,000 population) in 1990; **(B)** Incidence rate (per 100,000 population) in 2021; **(C)** Mortality rate (per 100,000 population) in 1990; **(D)** Mortality rate (per 100,000 population) in 2021; **(E)** DALYs rate (per 100,000 population) in 1990; **(F)** DALYs Rate (per 100,000 population) rate in 2021.

As in 1990, the regions with the highest incidence rates among AYAs in 2021 were concentrated in parts of Asia and Africa (Figure 2). Specifically, Somalia (259.67 per 100,000 [95% UI: 182.21 to 334.84]) and the Democratic Republic of the Congo (243.31 per 100,000 [95%UI: 177.14 to 310.03]) had the highest rates, while the United States of America (4.038 per 100,000 [95%UI: 3.05 to 5.15]) had the lowest (Figure 2 and Supplementary Table 5). In comparison to 1990, most countries, except the United Kingdom, experienced varying degrees of decline in the incidence rate by 2021. Among these, Niue and Brunei Darussalam showed the most prominent reductions (Supplementary Table 5). In addition, the overall incidence and mortality rates in China decreased by 53.27 and 53.25%, respectively (Supplementary Table 5). The highest mortality rates from hepatitis B-related cirrhosis among AYAs were observed in Kiribati (6.04 per 100,000 [95% UI: 3.68 to 8.93]) and Cambodia (5.42 per 100,000 [95% UI: 3.19 to 8.40]) (Supplementary Table 5). In contrast, Nordic countries, such as Sweden (0.03 per 100,000 [95% UI: 0.02 to 0.04]) and Norway (0.04 per 100,000 [95% UI: 0.03 to 0.05]) had the lowest mortality rates (Figure 2 and Supplementary Table 5). Ukraine, the Russian Federation, and Belarus showed the most pronounced rise in mortality rates in 2021 compared to 1990 which was similarly reflected in DALYs (Supplementary Table 5).

### 3.5 The incidence, mortality, and DALYs rates of hepatitis B-related cirrhosis in different regions and SDI-level areas

Regarding the AAPC of incidence, all 21 regions worldwide declined from 1990 to 2021 (Table 2). While mortality rates decreased in most regions, they increased in Eastern Europe (AAPC = 5.50, 95% CI: 3.93 to 7.09, *p* < 0.001) and Central Asia (AAPC = 1.40, 95% CI: 0.75 to 2.05, *p* < 0.001). The DALYs trends mirrored those of the mortality rates (Table 2). Similar to 1990, the regions with the highest incidence rates were Central Sub-Saharan Africa (221.97 per 100,000 [95% UI: 163.70 to 281.87]) and Western Sub-Saharan Africa (174.89 per 100,000 [95% UI: 138.49 to 213.95]) in 2021 (Table 1). Meanwhile, these two regions also had the highest mortality rates, at 2.93 per 100,000 (95% UI: 1.84 to 4.32) and 2.59 per 100,000 (95% UI: 1.76 to 3.53), respectively (Table 1). Conversely, Western Europe and High-income North America had the lowest mortality rates at 0.10 per 100,000 (95% UI: 0.06 to 0.15) and 0.14 per 100,000 (95% UI: 0.11 to 0.20) (Table 1).

**Figure 3:**
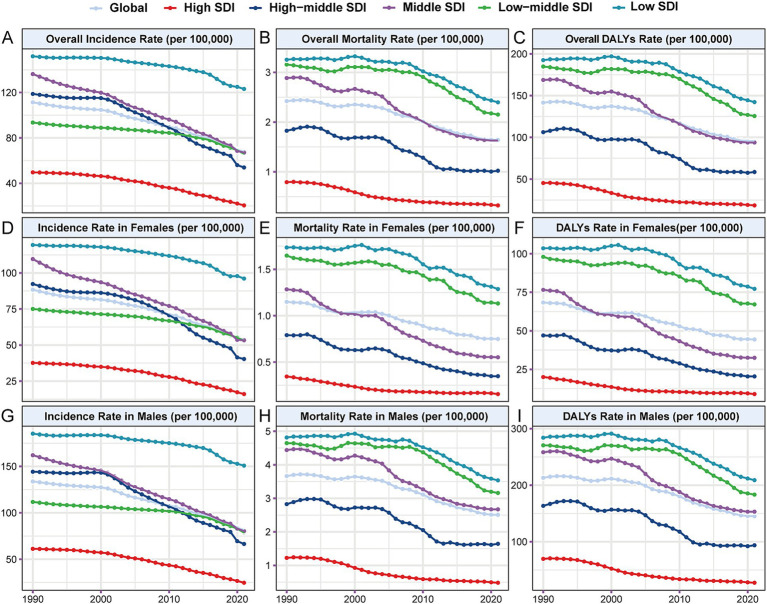
Trends of incidence, mortality, and DALYs rates (per 100,000 population) of Hepatitis B-related cirrhosis among AYAs from 1990 to 2021 at different SDI levels. **(A)** Overall incidence rate; **(B)** Overall mortality rate; **(C)** Overall DALYs rate; **(D)** Incidence rate in females; **(E)** Mortality rate in females; **(F)** DALYs rate in females; **(G)** Incidence rate in males; **(H)** Mortality rate in males; **(I)** DALYs rate in males. SDI: Sociodemographic Index.

**Figure 4:**
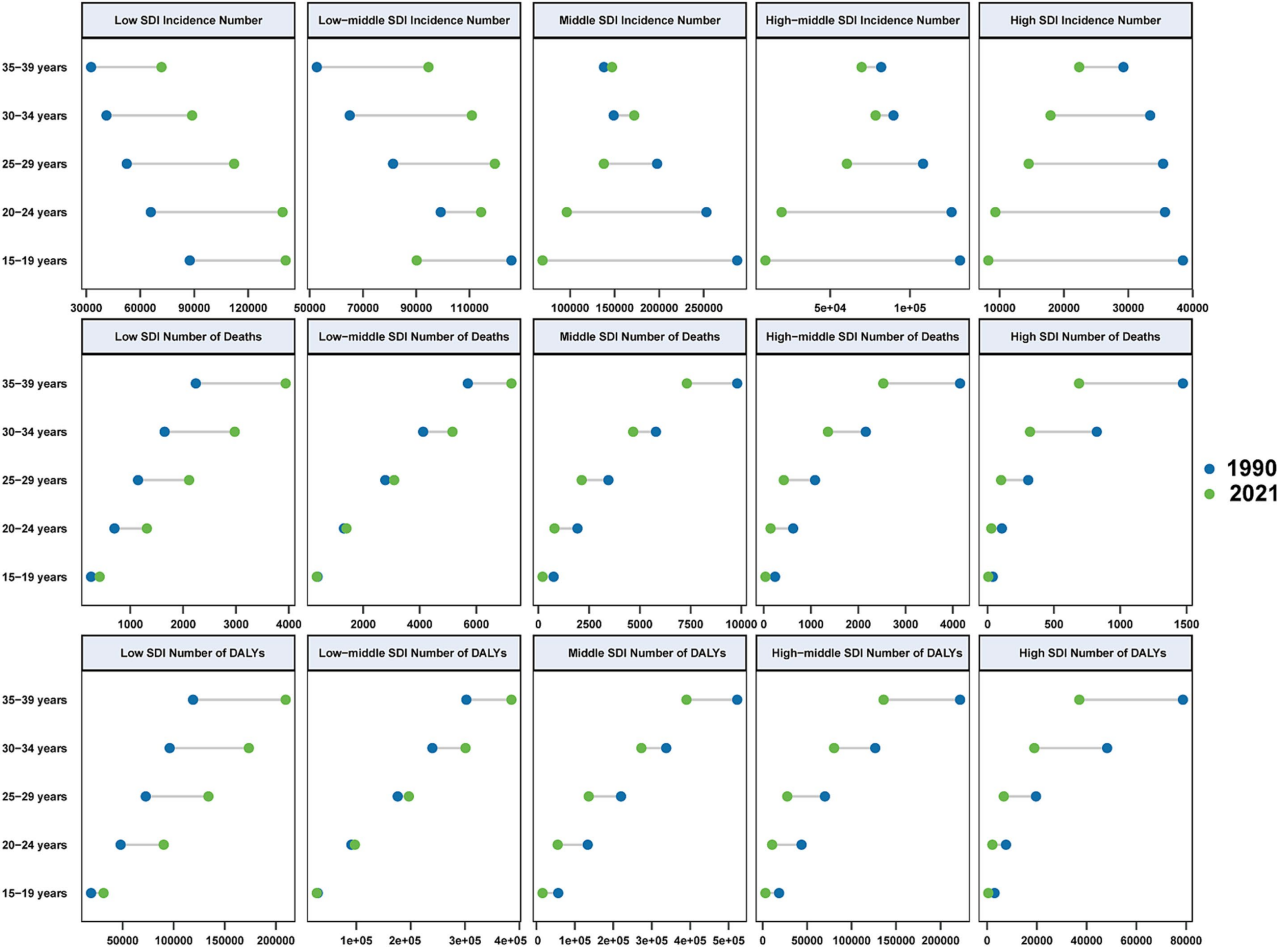
The numbers of incidences, deaths, and DALYs of Hepatitis B-related cirrhosis among AYAs in 1990 and 2021 by SDI level and age.

From 1990 to 2021, there was a consistent decline in incidence rates across all SDI levels, with a corresponding decrease in AAPC of incidence (Figure 3, Supplementary Figure 3, and Table 2). However, the burden of hepatitis B-related cirrhosis among AYAs varied significantly across different SDI-level areas (Table 1 and Figures 3, 4). Specifically, the highest incidence rate in 2021 was observed in low-SDI areas (123.11 per 100,000 [95% UI: 97.28 to 150.32]), while the lowest incidence rate was found in the high-SDI areas (20.51 per 100,000 [95% UI: 16.79 to 25.07]) (Figure 3 and Table 1). Similarly, there were notable disparities in the rates of mortality and DALYs across different SDI levels (Figures 3, 4). Notably, compared with 1990, the numbers of incidences, deaths, and DALYs across all age groups significantly increased by 2021 in low-and low-middle SDI areas (Figure 4).

## 3.6 BAPC model prediction of the incidence, mortality, and DALYs of hepatitis B-related cirrhosis from 2022 to 2035

**Figure 5:**
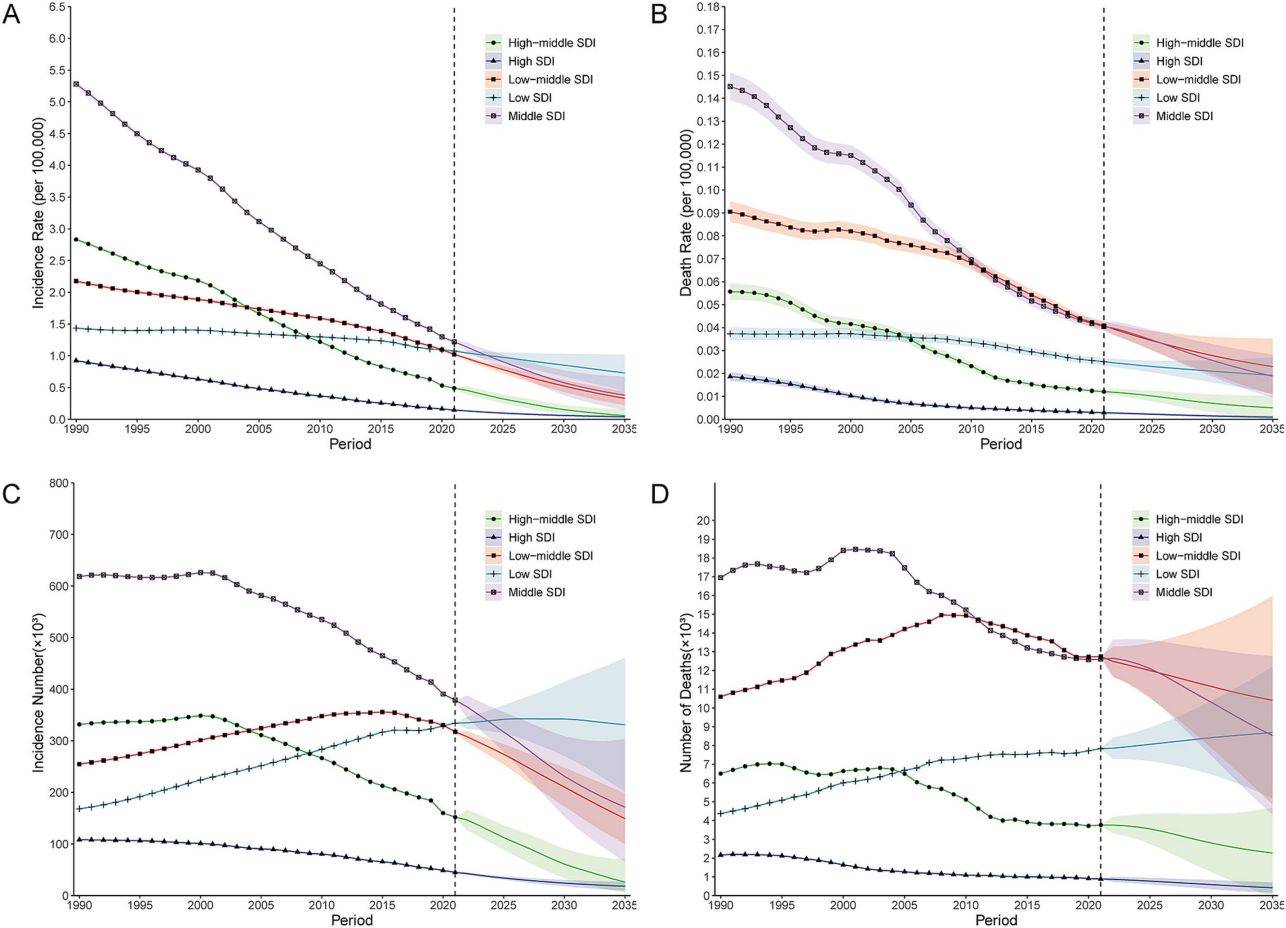
BAPC model prediction of incidences and deaths of Hepatitis B-related cirrhosis among AYAs from 2022 to 2035 at different SDI levels. **(A)** Prediction of incidence rate (per 100,000 population); **(B)** Prediction of mortality rate (per 100,000 population); **(C)** Prediction of incidence number (×10^3^); **(D)** Prediction of number of deaths (×10^3^). BAPC, Bayesian age-period-cohort.

The BAPC model predicted a continued decline in the global incidence, mortality, and DALYs rates of hepatitis B-related cirrhosis among AYAs from 2022 to 2035 (Supplementary Figures 4–6 and Supplementary Table 6). The overall numbers of incidences and deaths are expected to decline with predicted values of 643.44 × 10^3^ (95% CI: 303.08 × 10^3^ to 983.80 × 10^3^) and 31.68 × 10^3^ (95% CI: 18.88 × 10^3^ to 44.47 × 10^3^) by 2035, respectively (Supplementary Figures 7–9 and Supplementary Table 7). Furthermore, the incidence and mortality rates at different SDI levels are expected to decrease, reducing disparities across various SDI level areas (Figures 5A,B and Supplementary Table 8). Notably, the numbers of incidences and deaths in low-SDI areas are projected to continue increasing (Figures 5C,D). Specifically, the BAPC model predicted that the incidence number in low-SDI areas will rise to 331.02 × 10^3^ (95% CI: 201.39 × 10^3^ to 460.65 × 10^3^) by 2035 (Supplementary Table 9). Additionally, the estimated number of deaths in low-SDI areas is expected to increase to 8.69 × 10^3^ (95% CI: 5.18 × 10^3^ to 12.20 × 10^3^) by 2035 (Supplementary Table 9).

## 4 Discussion

In this study, we comprehensively explored the incidence, mortality, and DALYs rates of hepatitis B-related cirrhosis among AYAs (15–39 years) at the global, regional, and national levels for the first time using data from the GBD 2021 database. The study revealed significant changes in global incidence, mortality, and DALYs over three decades, with predictions extending to 2035.

Our finding revealed a downward trend in the incidence, mortality, and DALYs rates of hepatitis B among AYAs worldwide from 1990 to 2021. These findings align with previous studies on the global burden of hepatitis B and cirrhosis (1, 3, 9). Of particular interest is the observation that the rates of mortality and DALYs reached a low point in 1997 according to the joinpoint model. This decline may be attributed to the initial promotion of the hepatitis B vaccine and vertical transmission prevention strategies in the early 1990s (26). This resulted in a significant decline in the short-term mortality rate by reducing new infections and vertical transmission. Furthermore, we noted a slower decline in the global mortality and DALYs rates in 2019. According to WHO data, the estimated deaths from viral hepatitis increased from 1.1 million in 2019 to 1.3 million in 2022, with 83% of deaths attributed to hepatitis B. This increase may be related to the sharp decline in CHB consultations, testing, and treatment rates during the COVID-19 pandemic (27, 28).

The data revealed disparities in the burden of hepatitis B-related cirrhosis among genders and age groups. It was observed that males consistently experienced higher rates of incidence and mortality than females, which may be related to higher alcohol consumption, increased occupational exposure risks, and differences in sex hormone levels (7, 29). These findings highlight the need for sex-specific public health interventions to address these disparities. Furthermore, the significant decline in incidence rates among adolescents (15–19 years) underscores the effectiveness of enhanced vaccination programs, particularly the vaccine catch-up program (30), as well as increased awareness of hepatitis B prevention and transmission (31).

However, the incidence number of HBV-related cirrhosis among AYAs (30–39 years) increased slightly, especially in low-and low-middle SDI areas. Our findings expand our global perspective on the prevention and control of hepatitis B: challenges, achievements, and pathways to its elimination by 2030. This is concerning because individuals aged 30–39 are at a critical stage in their social and economic life, and their health status significantly impacts their families and society. The most important reason for the slight increase in the incidence of HBV-related cirrhosis among AYAs (30–39 years) is the low proportion of children under 5 years and 5–19 years who are protected by the hepatitis B vaccine (32). Despite expanded newborn hepatitis B vaccination programs, approximately 28% of children under age 5 years and 47% of children aged 5–19 years remain unprotected from hepatitis B in low-and low-middle SDI areas (32). Low health awareness may lead to infrequent HBV screening, while unhealthy habits like drinking and smoking can accelerate CHB progression to cirrhosis (33). In addition, long-term antiviral treatment may be particularly challenging in this age group (34). This highlights the need for targeted interventions and enhanced surveillance to prevent CHB progression.

Despite the overall global decline, this study revealed substantial regional disparities in the incidence and mortality rates of hepatitis B-related cirrhosis. Several African countries, such as Somalia and the Democratic Republic of the Congo, had the highest incidence rates, while Kiribati and Cambodia had the highest mortality rates in the Western Pacific region. In contrast, the United States and Nordic countries exhibited significantly lower incidence and mortality rates. This result was inseparable from the local hepatitis B prevention and control policies. According to WHO data, Africa accounts for 63% of new HBV infections; however, only 18% of newborns in the region had received the hepatitis B birth dose vaccine. The Western Pacific region accounts for 47% of HBV-related deaths, but treatment coverage remains low (4). In addition, a study on hepatitis B prevention and control policies showed that Somalia had the lowest hepatitis B prevention and control policy score (35).

China is a populous country, and the incidence and mortality rates of hepatitis B-related cirrhosis among AYAs have declined significantly since 1990. Since the early 1990s, China has included hepatitis B vaccines in its immunization management plan, actively promoted anti-HBV treatment, and expanded the indications for antiviral treatment of CHB (36). This demonstrates that the hepatitis B vaccine and antiviral drugs play vital roles in reducing the incidence of hepatitis B-related cirrhosis. Furthermore, the variance and mutation rates of the HBV genotypes are important influencing factors. For example, hepatitis B virus genotype A is primarily distributed in Europe, America, and Africa, while Genotypes B and C are common in Asia, with genotype C being predominant in East Asia and Southeast Asia (37). Notably, genotype C exhibits a high mutation rate increasing susceptibility to cirrhosis and liver cancer (38). Moreover, patients with genotypes A and B showed a better response to interferon treatment than those with genotype C (37, 38). Furthermore, there has been a significant increase in the mortality rate in Eastern Europe, particularly in Ukraine and the Russian Federation. This upward trend may be linked to ongoing regional conflicts.

The significant correlation between the burden of cirrhosis and SDI score underscores the impact of socioeconomic factors on health outcomes. Low and low-middle SDI areas face significant challenges, including limited healthcare access, inadequate vaccination coverage, and insufficient resources for effective disease management. For example, financial protection was lower in the African Region, where only approximately one-third of the reporting countries provided viral hepatitis services free of charge (4). In addition, in some high-prevalence areas, such as sub-Saharan Africa, the incidence and mortality rates are high owing to poor sanitary conditions. This may be related to the lack of vaccination efforts and the risk of horizontal HBV transmission (39).

Liver cirrhosis and liver cancer associated with childhood-acquired CHB are leading causes of death among adults in low-and low-middle SDI areas. Based on recommendations of the WHO and multiple public health experiences (6, 30), the adoption of a nationwide free catch-up hepatitis B vaccination program for unvaccinated children and adolescents in low SDI areas, in addition to ongoing efforts to improve birth dose and newborn vaccination coverage, will be cost-saving and can generate significant population-wide health benefits.

Furthermore, enhanced financial support for public health development and increased access to antiviral treatment are essential for mitigating the future burden of hepatitis B-related cirrhosis in low-and middle-income countries. Anti-virus treatment can reduce the incidence of HBV-related complications and improve patient survival rate, and it is the most important treatment measure for chronic HBV infection (40). The WHO 2024 guidelines (6), which advocate increased HBV diagnosis rates, expanded treatment standards for patients with CHB, (including adolescents), and broader antiviral treatment indications, are crucial steps in mitigating the future burden of hepatitis B-related cirrhosis.

This study’s innovative application of the BAPC model provides robust and reliable predictions of future disease trends. These findings provide valuable information for the prevention and management of hepatitis B and cirrhosis in various countries and regions. However, our study had some limitations. First, the effects of alcohol consumption and dietary factors on hepatitis B-related cirrhosis among AYAs were not considered. Second, we did not differentiate between compensated and decompensated stages of cirrhosis. Additionally, although the GBD data are comprehensive, GBD data are based on estimates and corrections, which may introduce some deviations. Discrepancies in the quality of original data across different countries and territories, such as variations in diagnostic reporting and health monitoring levels, may lead to underreporting or overestimation. Nonetheless, using up-to-date data and advanced statistical methods, we ensured the reliability and reproducibility of our findings.

## 5 Conclusion

In summary, the overall burden of hepatitis B-related cirrhosis among AYAs significantly decreased from 1990 to 2021. However, there was a slight increase in incidence number among individuals aged 30–39 years. Furthermore, the substantial burden and projected increase in the numbers of incidences and deaths in low-SDI areas emphasize the need for sustained and targeted public health interventions. Enhancing financial support for public health, strengthening maternal and infant blockade of HBV infection, increasing antiviral coverage, and monitoring cirrhosis, especially in low-and middle-income countries, are crucial steps in mitigating the future burden of CHB and cirrhosis.

## Data Availability

Publicly available datasets were analyzed in this study. This data can be found at: https://ghdx.healthdata.org/gbd-2021.

## Footnotes

^1^ https://ghdx.healthdata.org/gbd-2021

^2^ https://vizhub.healthdata.org/gbd-results/

^3^ https://surveillance.cancer.gov/joinpoint/
