# Supplementary material for: Global temporal trends and projections of hepatitis B-related cirrhosis among adolescents and young adults from 1990 to 2035: an analysis based on the global burden of disease study 2021
Source: Front Public Health. 2025 Jan 8;12:1494388. doi: 10.3389/fpubh.2024.1494388 (PMC11751058; doi:10.3389/fpubh.2024.1494388)
Supplement: Supplementary file 1 [file Data_Sheet_1.DOCX]

Supplementary Materials

1. **Supplementary Figures**
   1. **Figure S1.** The global incidence, mortality, and DALYs rates (per 100,000 population) of Hepatitis B-related cirrhosis among AYAs in 1990 and 2021 by gender and age.
   2. **Figure S2.** The global numbers of incidences, deaths, and DALYs of Hepatitis B-related cirrhosis among AYAs in 1990 and 2021 by gender and age.
   3. **Figure S3.** The incidence, mortality, and DALYs rates (per 100,000 population) of Hepatitis B-related cirrhosis among AYAs in 1990 and 2021 by SDI level and age.
   4. **Figure S4.** Prediction of the global incidence rate (per 100,000 population) by gender from 2022 to 2035.
   5. **Figure S5.** Prediction of the global mortality rate (per 100,000 population) by gender from 2022 to 2035.
   6. **Figure S6.** Prediction of the global DALYs rate (per 100,000 population) by gender from 2022 to 2035.
   7. **Figure S7.** Prediction of the global incidence number (×10^3^) from 2022 to 2035.
   8. **Figure S8.** Prediction of the global number of deaths (×10^3^) from 2022 to 2035.
   9. **Figure S9.** Prediction of the global number of DALYs from 2022 to 2035.
2. **Supplementary Tables**
   1. **Supplementary Table 1.** SDI values and levels at the global, regional, and national levels.
   2. **Supplementary Table 2.** The join-point regression results of incidence rate among AYAs from 1990-2021 at global and regional levels.
   3. **Supplementary Table 3.** The join-point regression results of the mortality rate among AYAs from 1990-2021 at global and regional levels.
   4. **Supplementary Table 4.** The join-point regression results of DALYs rate among AYAs from 1990-2021 at global and regional levels.
   5. **Supplementary Table 5.** The Incidence, death, and DALYs rates in 1990 and 2021 in 204 countries and territories.
   6. **Supplementary Table 6.** BAPC model prediction of global incidence, mortality, and DALYs rates from 2022 to 2035.
   7. **Supplementary Table 7.** BAPC model prediction of numbers of incidence, deaths, and DALYs from 2022 to 2035
   8. **Supplementary Table 8.** BAPC model prediction of incidence and mortality rates from 2022 to 2035 at different SDI levels.
   9. **Supplementary Table 9.** BAPC model prediction of numbers of incidences and deaths from 2022 to 2035 at different SDI levels.


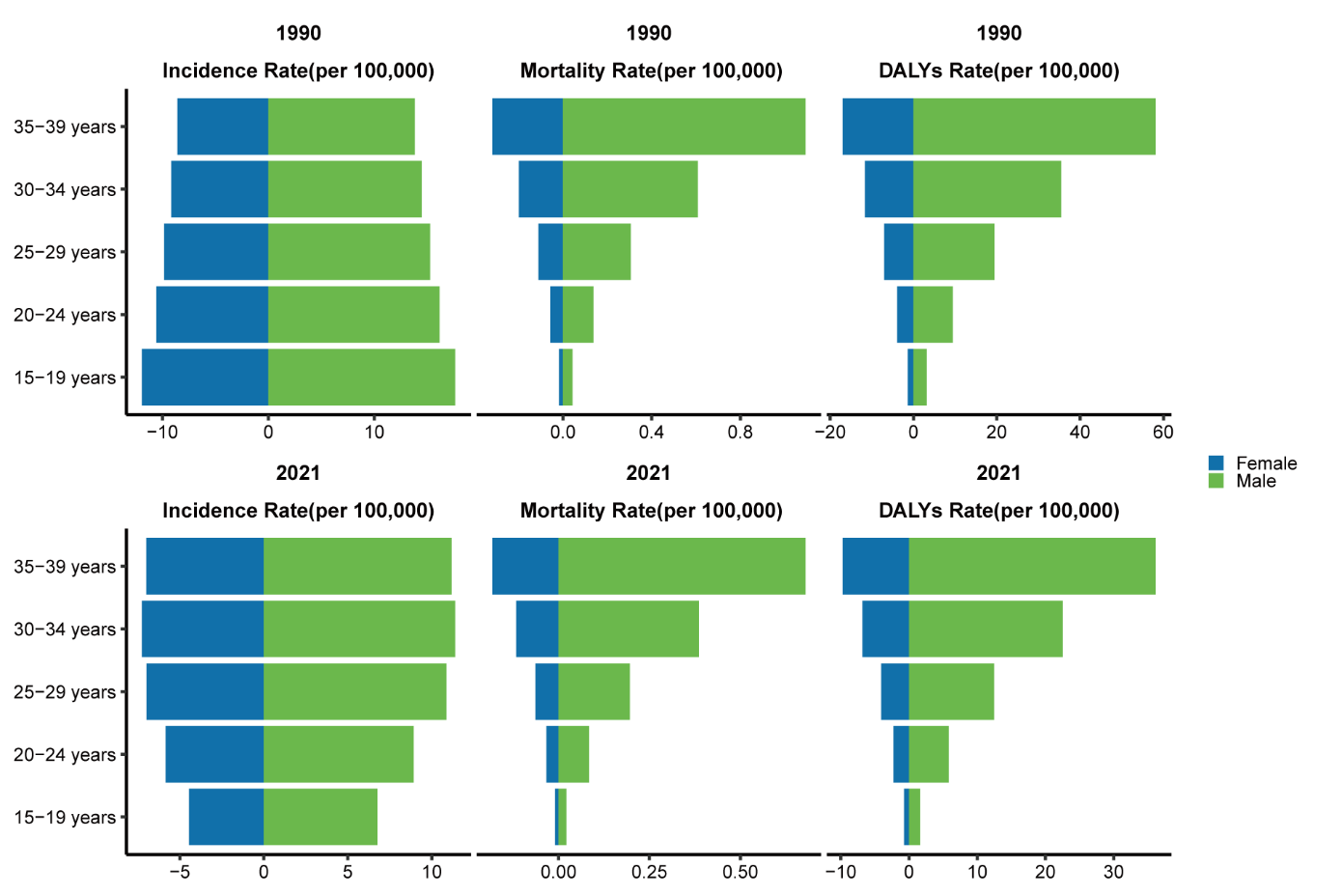


**Figure S1.** The global incidence, mortality, and DALYs rates (per 100,000 population) of Hepatitis B-related cirrhosis among AYAs in 1990 and 2021 by gender and age.

**
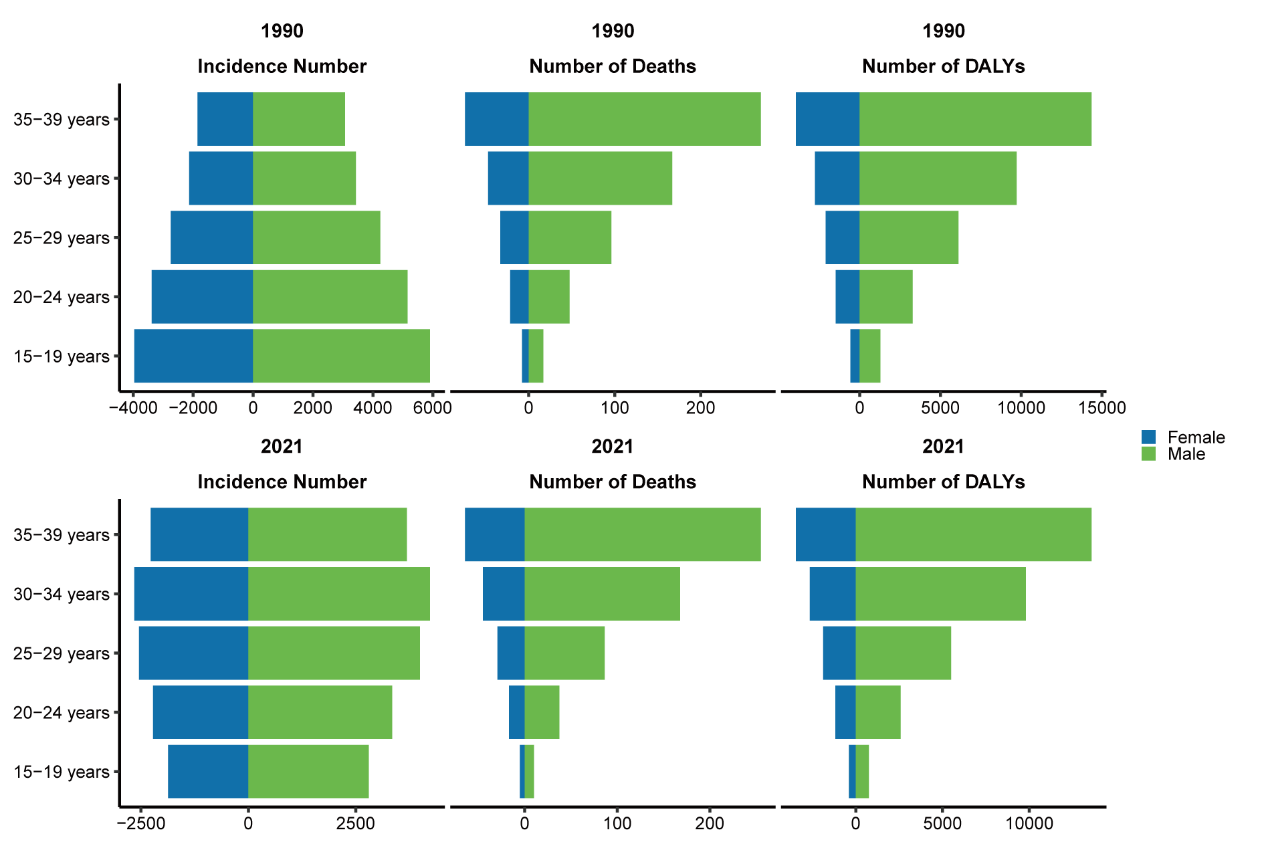
**

**Figure S2.** The global numbers of incidences, deaths, and DALYs of Hepatitis B-related cirrhosis among AYAs in 1990 and 2021 by gender and age.

**
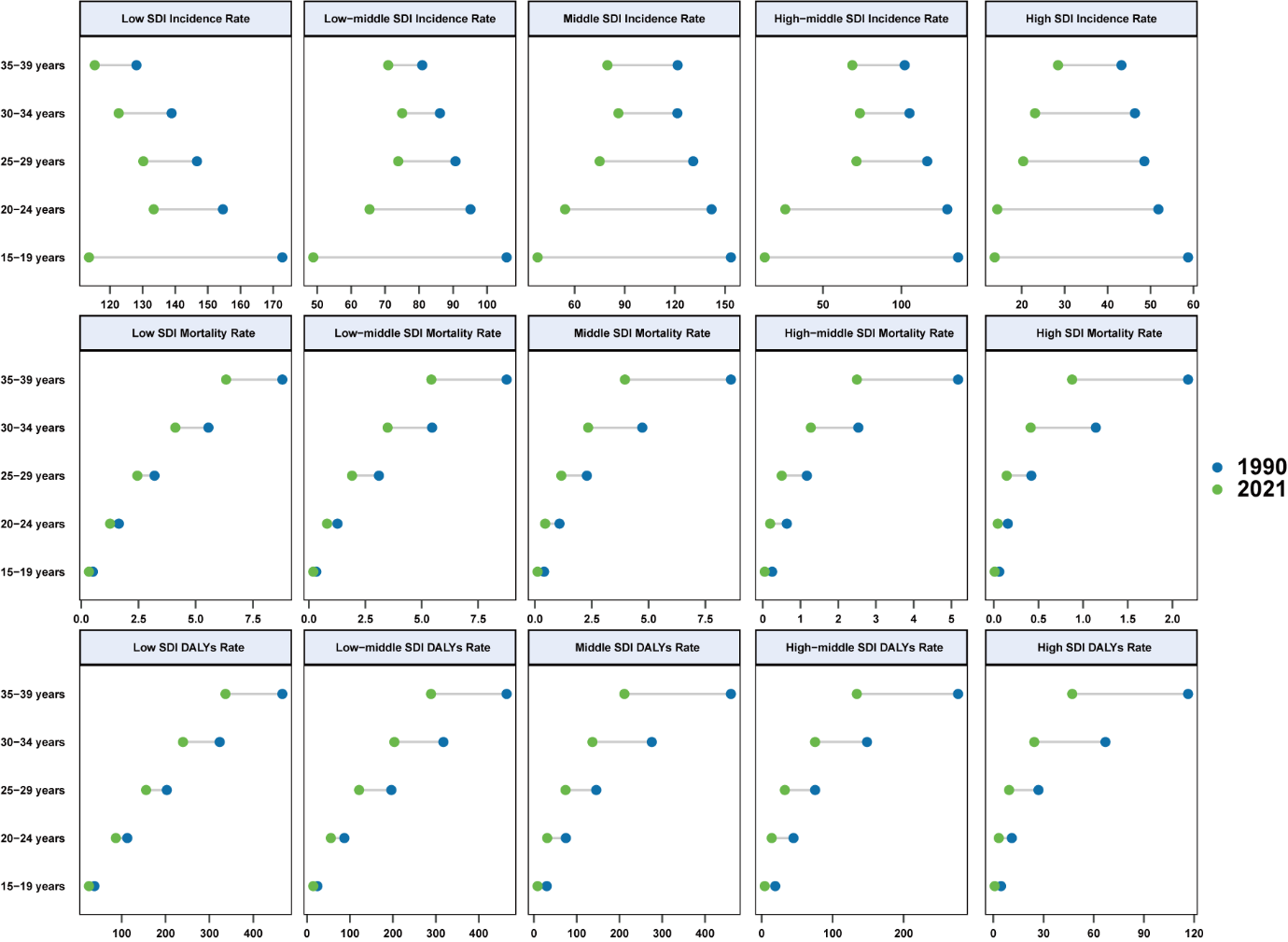
**

**Figure S3.** The incidence, mortality, and DALYs rates (per 100,000 population) of Hepatitis B-related cirrhosis among AYAs in 1990 and 2021 by SDI level and age.

**
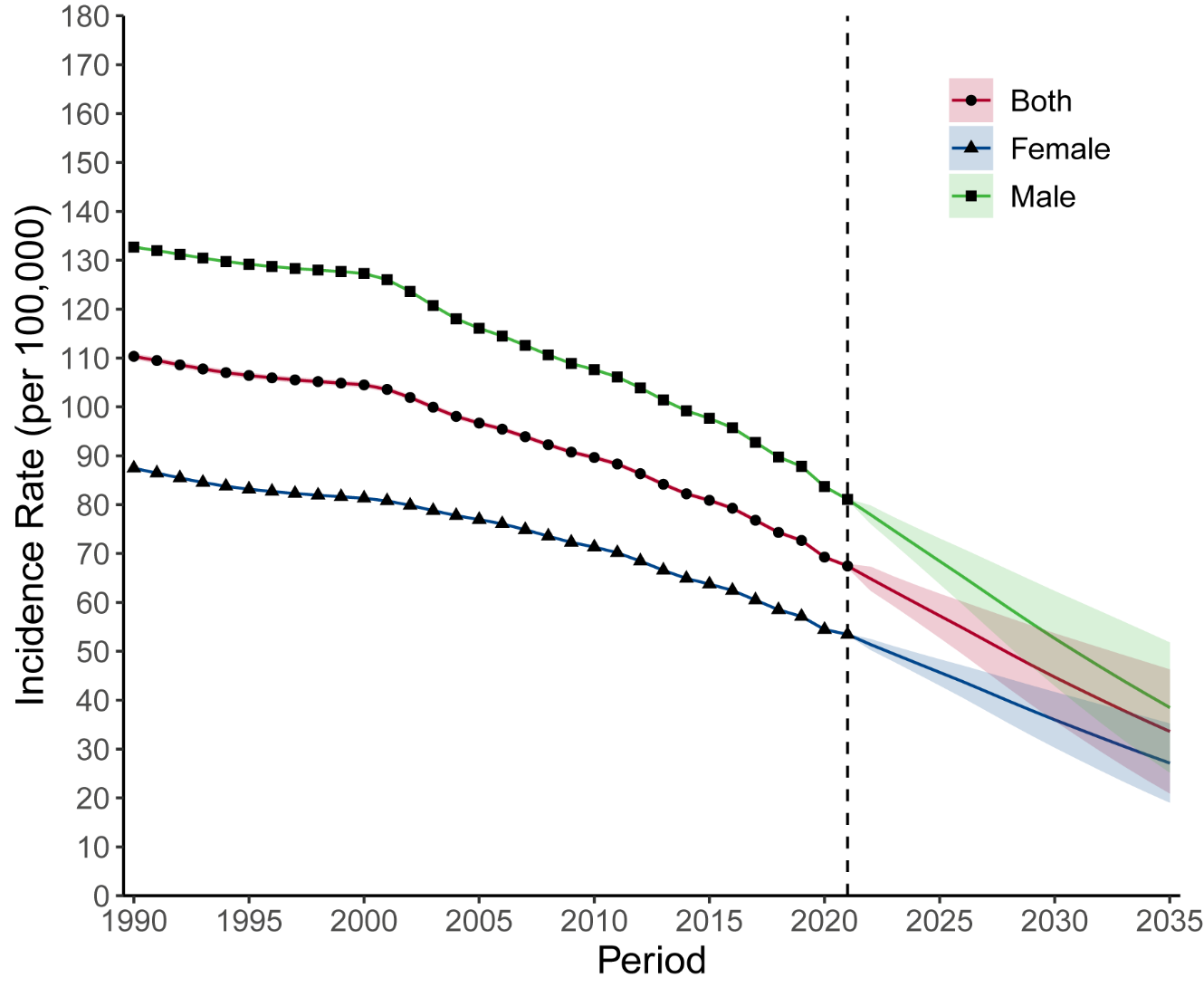
**

**Figure S4.** Prediction of the global incidence rate (per 100,000 population) by gender from 2022 to 2035.

**
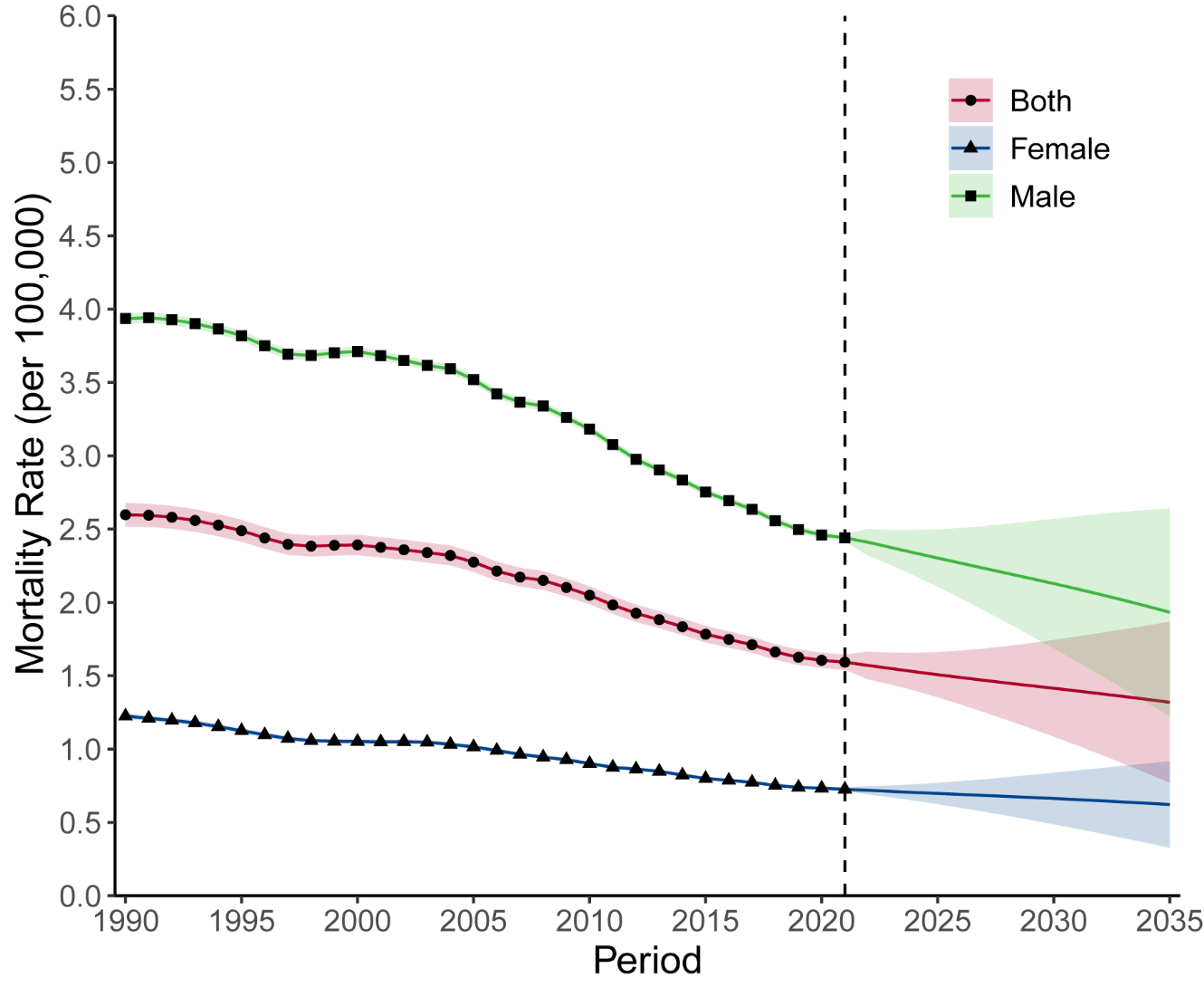
**

**Figure S5.** Prediction of the global mortality rate (per 100,000 population) by gender from 2022 to 2035.

**
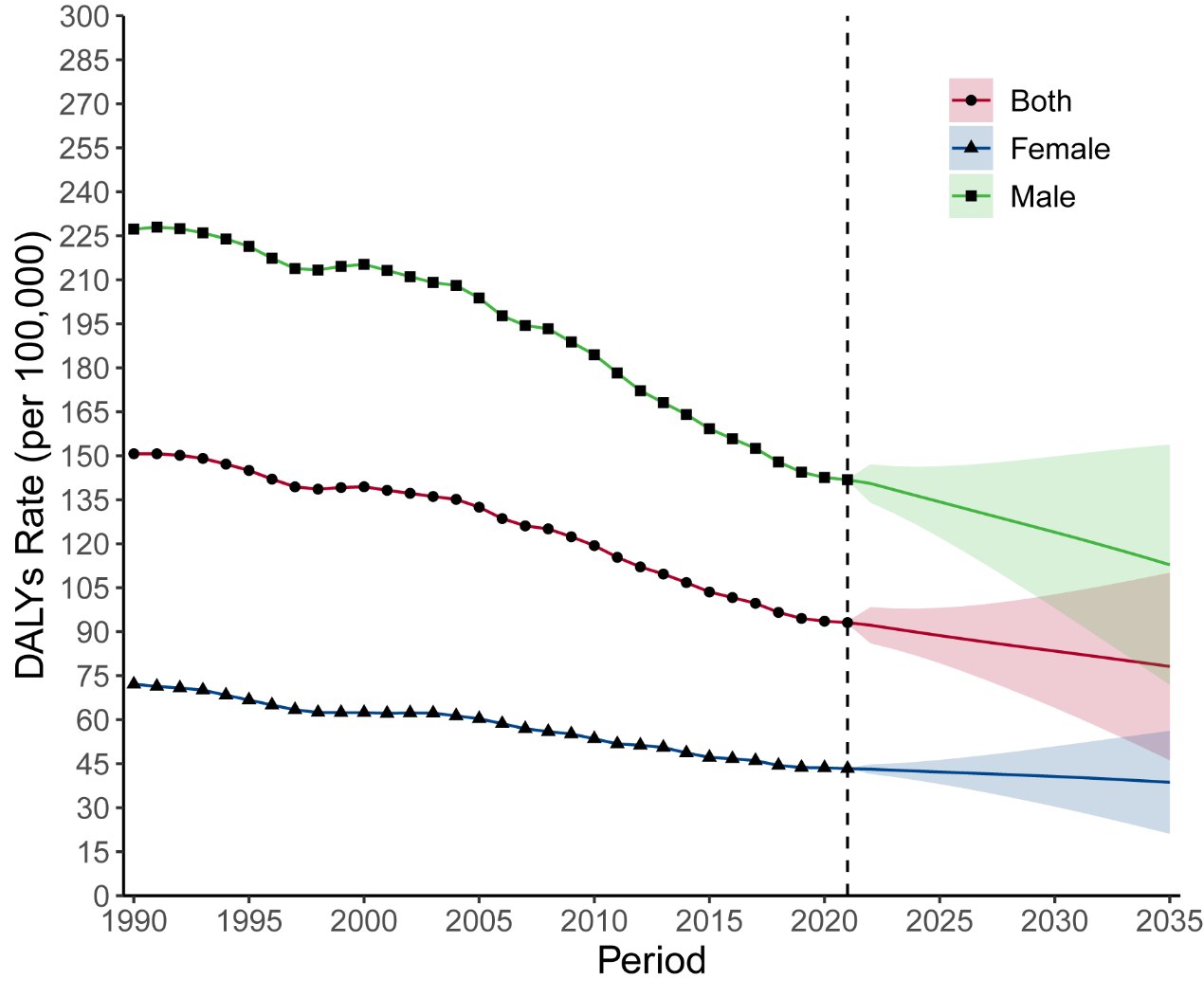
**

**Figure S6.** Prediction of the global DALYs rate (per 100,000 population) by gender from 2022 to 2035.

**
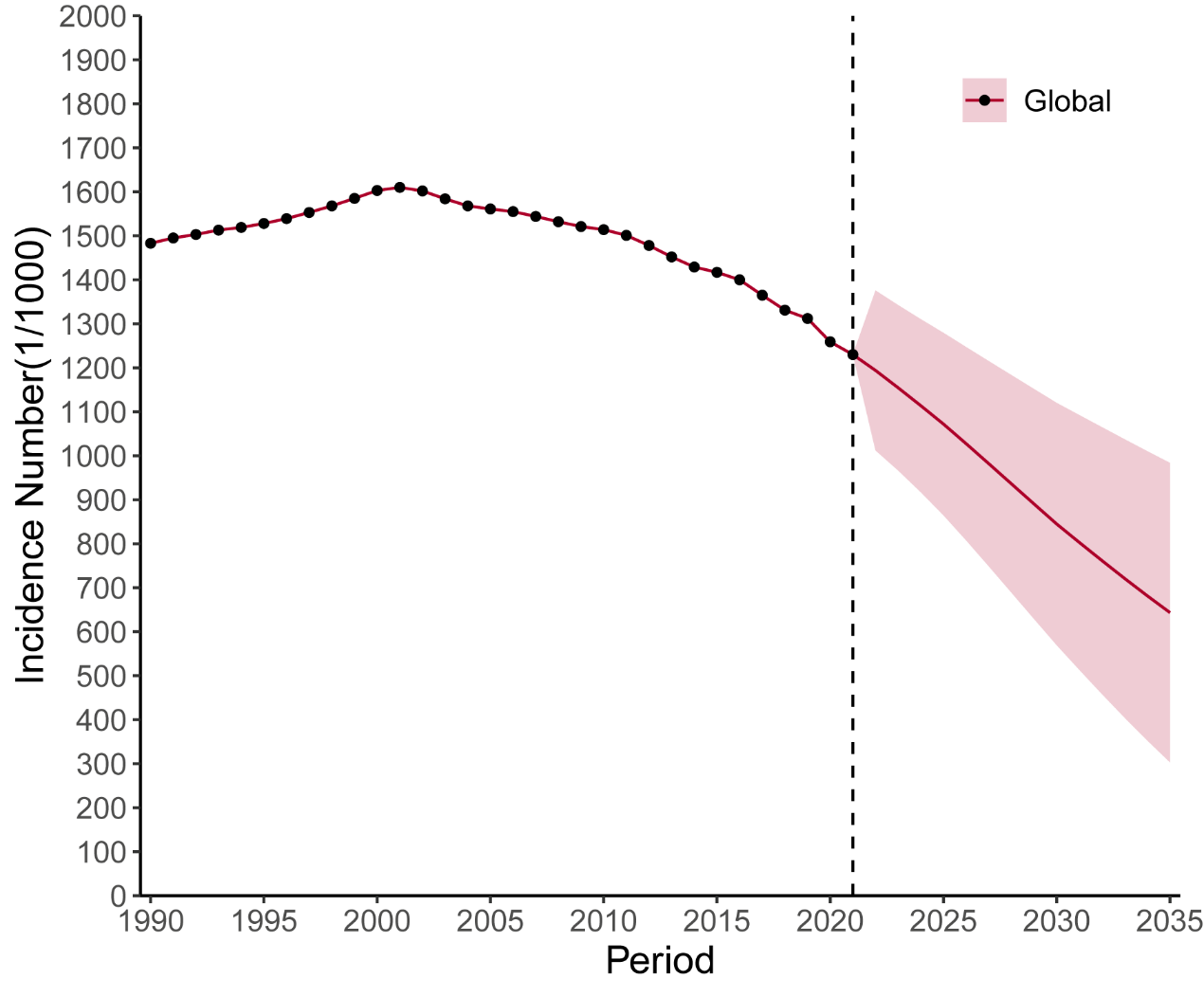
**

**Figure S7.** Prediction of the global incidence number (×10^3^) from 2022 to 2035.

**
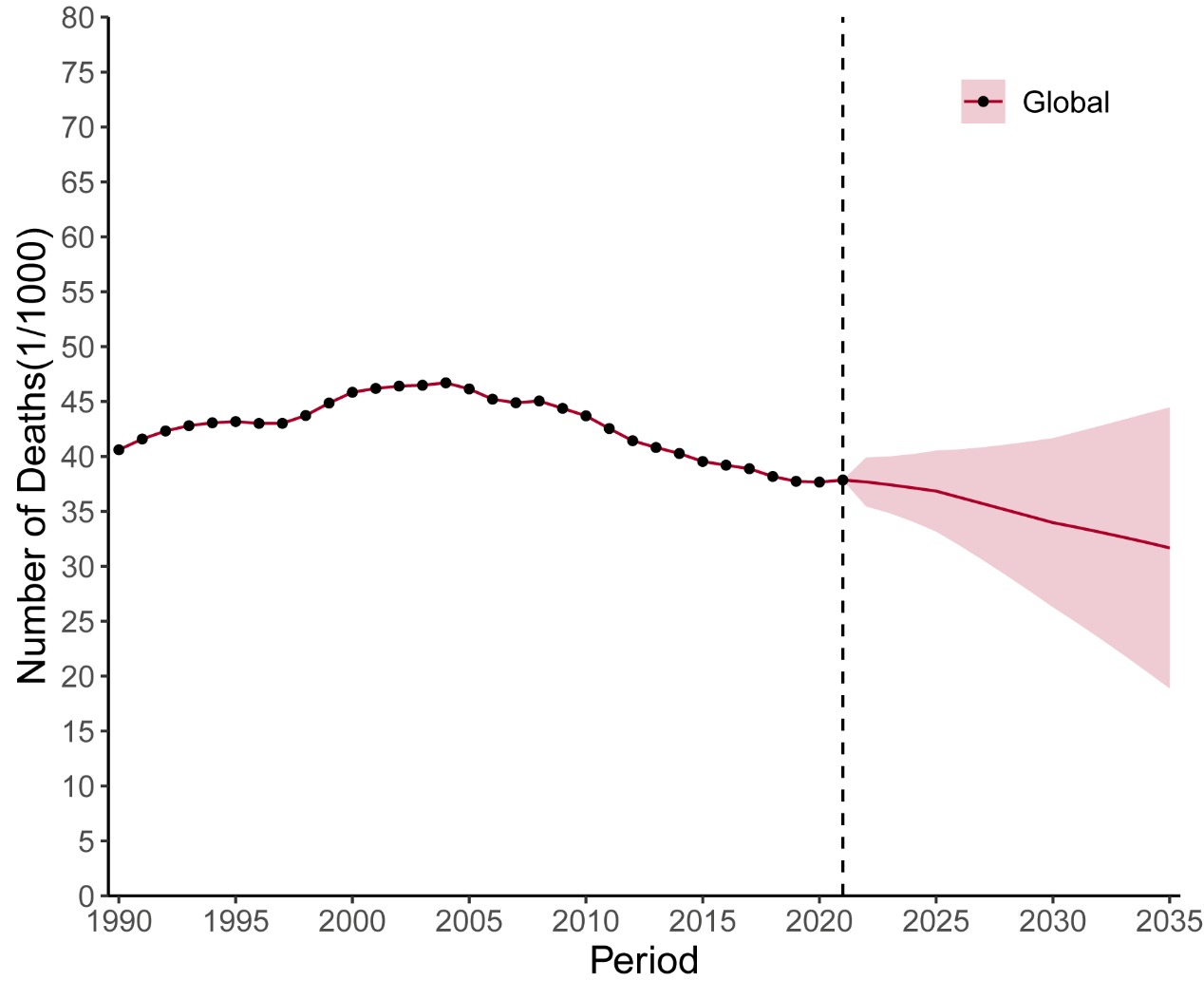
**

**Figure S8.** Prediction of the global number of deaths (×10^3^) from 2022 to 2035.

**
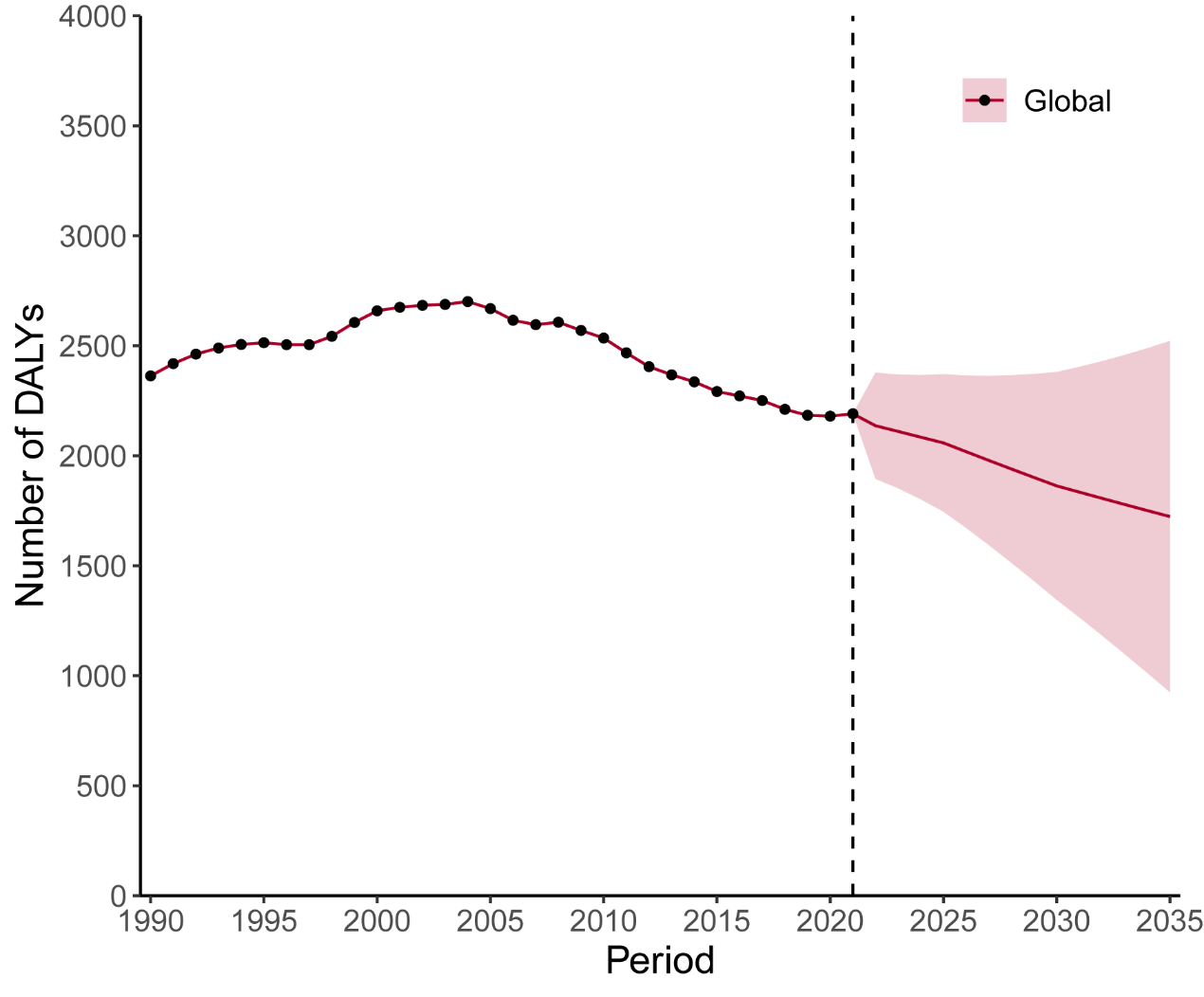
**

**Figure S9.** Prediction of the global number of DALYs from 2022 to 2035.
